# Detection of Simulated Fukushima Daichii Fuel Debris Using a Remotely Operated Vehicle at the Naraha Test Facility

**DOI:** 10.3390/s19204602

**Published:** 2019-10-22

**Authors:** Matthew Nancekievill, Jose Espinosa, Simon Watson, Barry Lennox, Ashley Jones, Malcolm J. Joyce, Jun-ichi Katakura, Keisuke Okumura, So Kamada, Michio Katoh, Kazuya Nishimura

**Affiliations:** 1 School of Electrical and Electronics Engineering, University of Manchester, Manchester M1 3BB, UK; joseluis.espinosamendoza@postgrad.manchester.ac.uk (J.E.); simon.watson@manchester.ac.uk (S.W.); barry.lennox@manchester.ac.uk (B.L.); 2 Department of Engineering, Lancaster University, Lancaster LA1 4YW, UK; a.jones1@lancaster.ac.uk (A.J.); m.joyce@lancaster.ac.uk (M.J.J.); 3 Department of Nuclear System Safety Engineering, Nagaoka University of Technology, Nagaoka 940-2188, Japan; j_katakura@vos.nagaokaut.ac.jp; 4 Japan Atomic Energy Agency, Iwaki 970-8026, Japan; okumura.keisuke@jaea.go.jp; 5 Marine Risk Assessment Department, National Maritime Research Institute, Mitaka 181-0004, Japan; kamada-s@nmri.go.jp (S.K.); kato-m@nmri.go.jp (M.K.); k_nishimura@nmri.go.jp (K.N.)

**Keywords:** robotics, nuclear characterization, underwater, submersible, ROV, 3D reconstruction, mapping, localization, vision

## Abstract

The use of robotics in harsh environments, such as nuclear decommissioning, has increased in recent years. Environments such as the Fukushima Daiichi accident site from 2011 and the Sellafield legacy ponds highlight the need for robotic systems capable of deployment in hazardous environments unsafe for human workers. To characterise these environments, it is important to develop robust and accurate localization systems that can be combined with mapping techniques to create 3D reconstructions of the unknown environment. This paper describes the development and experimental verification of a localization system for an underwater robot, which enabled the collection of sonar data to create 3D images of submerged simulated fuel debris. The system was demonstrated at the Naraha test facility, Fukushima prefecture, Japan. Using a camera with a bird’s-eye view of the simulated primary containment vessel, the 3D position and attitude of the robot was obtained using coloured LED markers (active markers) on the robot, landmarks on the test-rig (passive markers), and a depth sensor on the robot. The successful reconstruction of a 3D image has been created through use of a robot operating system (ROS) node in real-time.

## 1. Introduction

In 2011, a tsunami struck the coast of Japan which caused the Fukushima Daiichi nuclear power plant back-up generators to fail. The loss of power meant that the cooling systems failed, leading to the fuel melting. As the fuel melted, it fell to the bottom of the reactor pressure vessels (RPVs), then melted through to the base of the pedestal within the primary containment vessel (PCV) [1].

Areas within the Fukushima nuclear power plant are now classified as being hazardous. High radiation levels in the region of 10 Gy·h^−1^ have been recorded [1,2], with higher dose-rates predicted further into the power plant. The area is also very compact with debris strewn across many areas making access very difficult. There have already been examples of robots either failing due to radiation [3], or becoming entangled and stuck [2] within Fukushima. It is therefore imperative to develop robust systems capable of characterising the environment and returning the required data [4].

To enable decommissioning of the site, the exact location of the remaining fuel debris must be found and the fuel characterised, both physically and radiologically. The fuel debris is currently submerged to keep the remaining fuel cool and continually circulated to enable the water to remain cool. Due to this circulation of water, there are consistent, albeit slow, currents within the water, dust, rust, and sludge, with poor turbidity and lighting conditions. Any system deployed within the environment must have a small form-factor, operate in variable and unknown environments and offer safe control through fast refresh-rates of position. These constraints are also true of nuclear decommissioning sites such as the Sellafield legacy ponds for nuclear fuel storage.

A number of submersible robots have been developed to characterise the submerged environments. These include the “Little Sunfish” [5] developed by Toshiba, Hitachi’s swimming robot [6], and the University of Manchester’s AVEXIS${}^{\mathrm{TM}}$ robot [7]. Plant operators must be able to localise the remotely operated vehicle (ROV), which will enable the operators to know the real-world position of fuel debris and nuclear waste that is characterised by the ROV. 3D reconstructions of the environment can then be made with radiological data over-laid onto the model.

### Naraha Technology Demonstration Facility

The ability of our ROV and sensor to complete this task was experimentally verified through a technical demonstration at the Naraha test facility, Japan. The test facility replicates the conditions (i.e., the shape of the environment and depth) of the reactors at the Fukushima accident site in Japan. The Naraha Tank is a 5 m wide by 5 m tall cylinder used to test platforms that might be deployed in the Fukushima reactors. Due to the available testing facility, our proposed solution had to be simple enough to deploy in a short period of time and robust to unforeseen difficulties arising out of development of the technology in the UK before demonstration in Japan.

**Figure 1:**
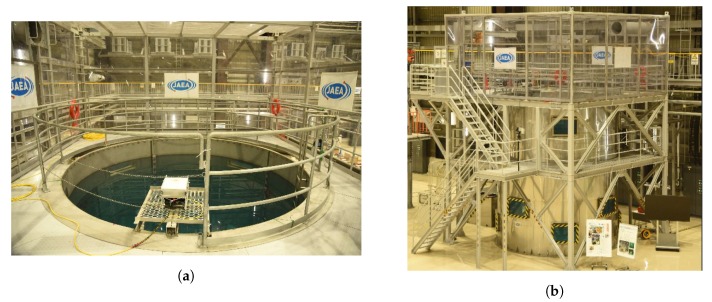
View of the access to the robot test pool can be seen in (**a**), and overall view of the robot test pool showing the side view windows and the access to the pool can be seen in (**b**) [8].

Figure 1a,b outline the Naraha Tank in which the AVEXIS${}^{\mathrm{TM}}$ was deployed. Figure 1a is the working space on the top of the tank with Figure 1b highlighting the windows around the outside to observe the ROV during operation.

**Figure 2:**
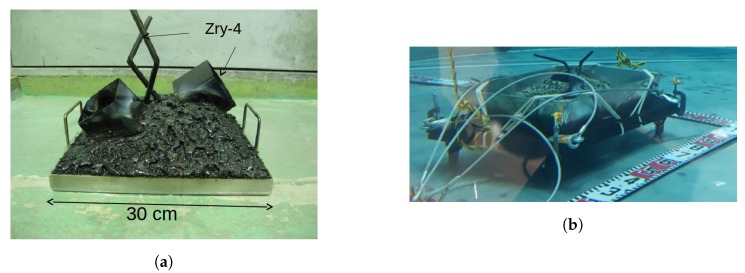
Simulated debris placed at the bottom of the tank as seen in-air, (**a**), before simulated sludge was added and from a submerged camera after deployment with sludge, (**b**).

Simulated fuel debris, as seen in Figure 2, was covered in simulated sludge (to simulate the sludge that has built-up in the PCV over the last seven years) and then lowered to the bottom of the tank. It was made up of various shapes and sizes of materials, indicative of the expected Fukushima environment.

## 2. Proposed Solution

The proposed solution to demonstrate the characterization of submerged nuclear fuel debris and waste, is to adapt The University of Manchester AVEXIS${}^{\mathrm{TM}}$ ROV to physically characterise the submerged environment through use of a mounted IMAGENEX 831L sonar [9]. This will then be combined with the vision localization system outlined in Section 3, that will deploy a camera above the working environment. A 3D reconstruction of the inside of the environment could then be created from this combined data that would highlight the location and physical dimensions of the fuel debris.

### 2.1. The AVEXIS${}^{\mathrm{TM}}$ Remotely Operated Vehicle

**Figure 3:**
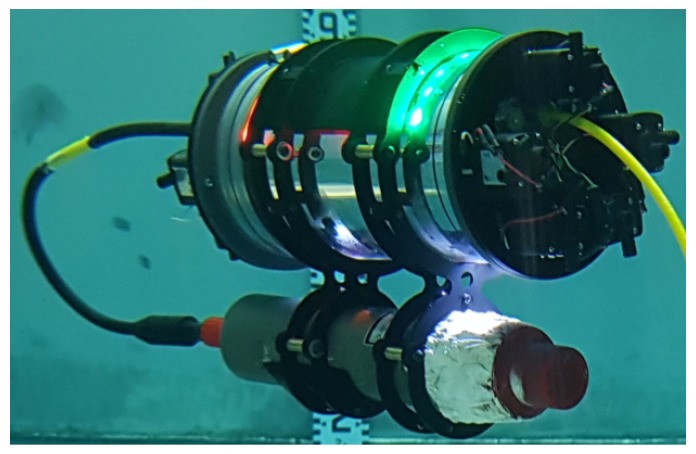
Mounting of the IMAGENEX 831l sonar beneath the AVEXIS${}^{\mathrm{TM}}$.

The AVEXIS${}^{\mathrm{TM}}$ ROV, that will be used as a research platform within this work, was developed in collaboration between the University of Manchester, the University of Lancaster and the Japan Atomic Energy Agency (JAEA), the National Maritime Research Institute (NMRI), and Nagaoka University of Technology in Japan. It is a 6 in diameter, 300 mm long, three degrees of freedom (d.o.f) ROV that is both powered and communicated with through a single twisted pair of wires. Five aquatic pumps on each end-cap propel the AVEXIS${}^{\mathrm{TM}}$ with neutral buoyancy maintained through the use of ballast. The AVEXIS${}^{\mathrm{TM}}$ has a payload of approximately 1.5 kg. Initially designed to aid in characterization of nuclear storage fuel tanks, it has been adapted for use within Fukushima. Figure 3 is an overview of the ROV during an experiment.

### 2.2. Underwater Localization

The majority of underwater localization systems are based on measuring the range between a speaker or acoustic modem and a hydrophone [10]. They can reach localization and communication distances up to 10 km or even more by hopping through a network of modems covering a large area [11]. Range-based acoustic positioning systems can achieve accuracies of less than half a metre when the environment conditions are ideal [12].

It is rare for environmental conditions to be ideal however, as acoustic systems are strongly affected by environmental factors such as the depth of water (shallow water decreases the performance), multipath interference due to cluttered or noisy environments (e.g., enclosed environments or seabed), pressure/temperature gradients, ambient noise, salinity, turbidity and, if the underwater vehicle (UV) is moving fast enough, motion-induced Doppler shift [13].

Alternative underwater localization technologies include electromagnetic (EM) [14], inertial [15], sonar [16], Lidar [17], visual simultaneous localization and mapping (SLAM), [17,18,19,20] and sonar SLAM [21,22,23]. Table 1 summarises and compares a range of underwater localization technologies and shows the importance of different characteristics of the working environment.

The requirements for the localization system to be deployed in this research include a highly accurate system, in an enclosed environment (less than 5 m in diameter and 5 m deep) offering moderate latency. Acoustic systems can be used effectively at long ranges, but their use in small confined spaces, such as those encountered at Fukushima Daiichi, is limited due to the multipath effect cause by the submerged structures and the environment boundaries. In the case of EM based systems, their working range is limited to approximately one metre before repeaters are required, while inertial systems are not reliable over long time periods, due to drift. Sonar systems requiring complex SLAM algorithms to accurately calculate position, struggle in enclosed featureless environments [24]. The proposed test facilities offer few features on the walls of the tank, reducing the accuracy of sonar SLAM based systems.

Visual SLAM techniques also struggle with featureless environments [25], reducing accuracy with loop-closure difficult in a repetitive cylindrical tank environment. Lidar systems are prohibitively big and expensive. The accuracy and latency of image-based systems are mainly limited by the resolution of the camera and visibility. The visibility of the proposed environment is known to be relatively clear due to constant recycling, therefore, a vision localization system was chosen for the inspection tasks outline in Section 1.

A vision-based localization system can be achieved using a camera mounted above the proposed environment to track the location of the ROV [26]. By combining the vision system with a pressure sensor, full 3D coordinates for the AVEXIS${}^{\mathrm{TM}}$ could be estimated. The target accuracy was approximately 10 cm. The accuracy of this system was predicted to be appropriate for the aim of stitching sonar slices together to develop a 3D reconstruction of the submerged environment.

Commercially available external vision-based localization systems are prohibitively expensive and are also not suitable for this particular use-case as they are optimised for air operation, rather than submerged, and they require multiple cameras and calibration processes not possible in the enclosed environment. Therefore, a new low-cost, external image-based localization system was developed, using one camera, for less than US$150.

The rest of this paper is structured as follows; Section 2 presents the proposed solution to the problem, Section 3 gives an overview of the localization system, and Section 4 describes the 3D reconstruction of data from the sonar. Experimental verification and results are described in Section 5 for the localization system, Section 6 outlines the technical demonstration at the Naraha demonstration facility, and Section 7 describes the results of the fully integrated system. Section 8 offers a discussion on the results, whilst Section 9 concludes the work.

## 3. External Vision Localization System (EVLS)

**Figure 4:**
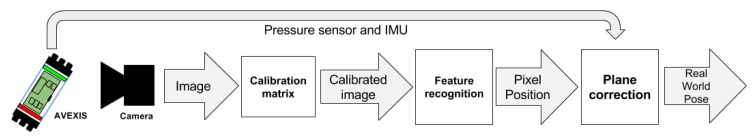
Flow chart of the localization system’s software steps.

The overall system diagram of the proposed external vision localization system (EVLS) is shown in Figure 4. The system initially obtains an image, then passes it through a calibration matrix to remove the lens induced distortions. Features of interest are then identified before the pixel position of the robot is translated to real-world coordinates. Depth and Inertial Measurement Unit (IMU) readings are gathered from the ROV to be taken into account during the translation to real-world coordinates.

**Figure 5:**
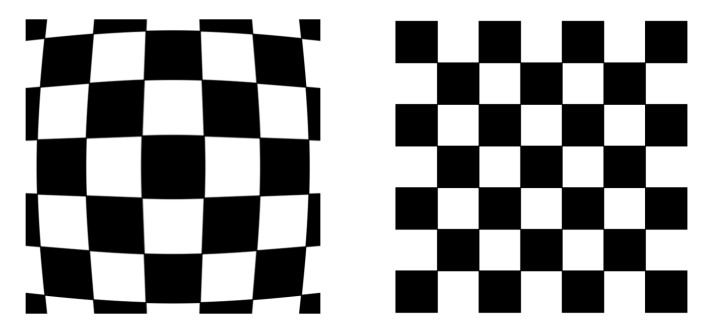
Demonstration of required calibration of fish-eye lens camera to “flatten” out the image to maintain consistent positions.

Calibration is required for lens distortion for most vision systems, but is of great importance in this work due to the use of a fish-eye lens camera and the increased radial distortion caused by the water interface. This camera was chosen to cover as large an area as possible to enable localization of the entire environment with one deployed camera. Therefore, the image returned from the camera must be calibrated as shown in Figure 5. Water distortion was not found to significantly increase the error of the proposed system to enable 3D reconstruction of the environment and so was not included in the calibration of the fish-eye lens.

The EVLS was required to be adaptable and applicable for deployment in any tank facility and to be able to view the entire work-space with no blind-spots. Each test tank has varied shapes, sizes, mounting locations and mounting angles for the camera. This camera angle, with respect to the water surface, must be as orthogonal as possible and not more than 25 degrees off centre to avoid the loss of line-of-sight of the submersible due to the internal reflection of water. This meant that the pixel to real-world co-ordinates in the field of view (FoV) of the camera system must be calibrated in each environment and deployment. Therefore, the system was required to track both passive and active markers. Eight passive landmark markers, which were placed around the inside of the tank for calibration of the ROV work-space at each new tank facility, and two active markers mounted on the AVEXIS${}^{\mathrm{TM}}$.

**Figure 6:**
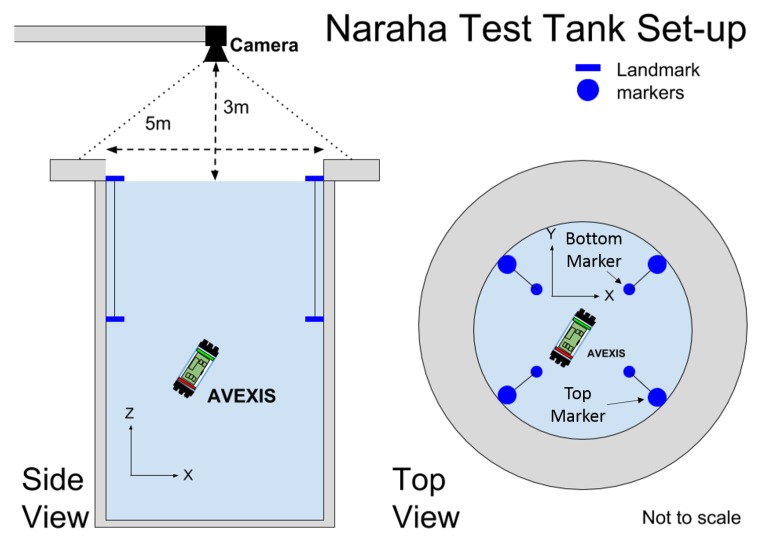
Example deployment of passive markers in the Naraha testing pool with the image localization system mounted.

The passive markers would not be required in a real-world environment, instead, characterization of the system in a representative environment would be required. The active markers consisted of a red LED strip and a green LED strip, which allowed the AVEXIS${}^{\mathrm{TM}}$ to be tracked for the full depth of the tank (5 m) and made the system robust against interference from the medium. The camera used had a FoV of 170${}^{\circ }$ and was selected so it could view the entire working environment and markers when positioned ≈3 m above the water surface as seen in Figure 6, which is a constraint of the test facility.

The multimedia effects of light passing between air and water have not been taken into account in this work. This is due to the proposed calibration of the system with respect to pre-determined markers. These markers enable the change in *x*, *y*, and *z* co-ordinates, within the marker space, to be determined and calibrated, allowing multimedia effects to be ignored. The camera’s position above the water at heights of 2 m and above also mean that the refraction of light is minimized, resulting in smaller errors over the system.

To translate the pixel position of the robot to real-world coordinates the system also makes use of the AVEXIS${}^{\mathrm{TM}}$’s on board pressure sensor [27], which was capable of determining its depth to an accuracy of less than 1 cm. This helped to correct for the refraction effect due to the medium interface of air to water and the perspective distortion dependant on the distance of the robot to the camera.

The localization system, the camera calibration, the feature recognition to identify the AVEXIS${}^{\mathrm{TM}}$, and the image rectification was achieved using OpenCV and its libraries using the Python${}^{\mathrm{TM}}$ programming language. The real-time 3D imaging used the robot operating system (ROS), with nodes programmed using the Python${}^{\mathrm{TM}}$ language.

### 3.1. Image Coordinates

**Figure 7:**
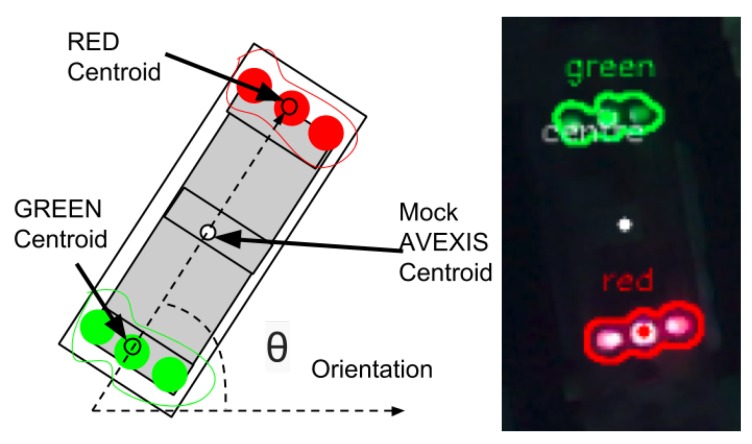
Image recognition of the pixel centroid of the AVEXIS${}^{\mathrm{TM}}$. Identifying the red and green LEDs and marking a centroid for each colour. The white point in the middle shows the AVEXIS${}^{\mathrm{TM}}$ centroid after determining the mean of the red and green centroids.

To estimate the position of the AVEXIS${}^{\mathrm{TM}}$ underwater, its pixel centroid (Pc), which is the position of the robot in the camera’s image, needs to be found. To achieve recognition of the robot, two coloured LED strips were installed on its top, one red and one green (as seen in Figure 7, which also identifies the Pc). The two different colours enable the yaw of the robot to be calculated as well as the (x,y) coordinates in relation to the camera.

To calculate Pc, the centroid of the red LED strip, Rpc, needs to be calculated. The green LED strip can then be found within a set distance from the red centroid, as the maximum distance between the centroids depends on the size of the robot. This is done to reduce the computing time required to find the robot in the image, as instead of re-scanning the image again, it only re-scans the pixels within the vicinity of the red marker. The radius of the vicinity depends on the maximum expected pixel size of the robot. This value is adjusted by the user of the system. This method reduces false positives caused by objects in the environment.

After finding the green marker, its centroid is calculated, Gpc. This information is used to calculate Pc as a simple average:

(1) (Rpc+Gpc)/2,

and then the orientation of the system:

(2) $$\theta =atan2(\left(Rpc_{y}-Gpc_{y}\right)/\left(Rpc_{x}-Gpc_{x}\right)).$$

These camera co-ordinates give a relative position of the robot within the FoV of the camera, the real-world co-ordinates now need to be found.

### 3.2. Real World Coordinates

A key challenge to overcome in the estimation of the real-world coordinates, Rc, is perspective distortion, which can manifest in two ways; depth distortion and camera misalignment. If the AVEXIS${}^{\mathrm{TM}}$ is held at a constant (*x*,*y*) coordinate, but it changes its depth, its (*x*,*y*) pixel position in the image will vary (depth distortion). If the camera FoV is off-centre, it will not be normal to the working environment, so the transformations are not direct (camera misalignment).

To overcome the distortions, circular, passive landmark markers of known size were distributed in the test tank. Eight markers were placed at known positions in the working environment. Four of these markers were positioned on top of the water surface, $P_{1-4}$ and the other four, $P_{1-4}^{\prime }$, at a depth of *D*. *D* can be any distance that fits inside the tank in a straight vertical line from the top markers to the bottom ones, as long as they can be seen by the camera.

Once the camera is positioned, the pixel position of the markers is manually introduced to the localization system. If the camera is moved, the pixel position of the markers needs to be re-introduced.

**Figure 8:**
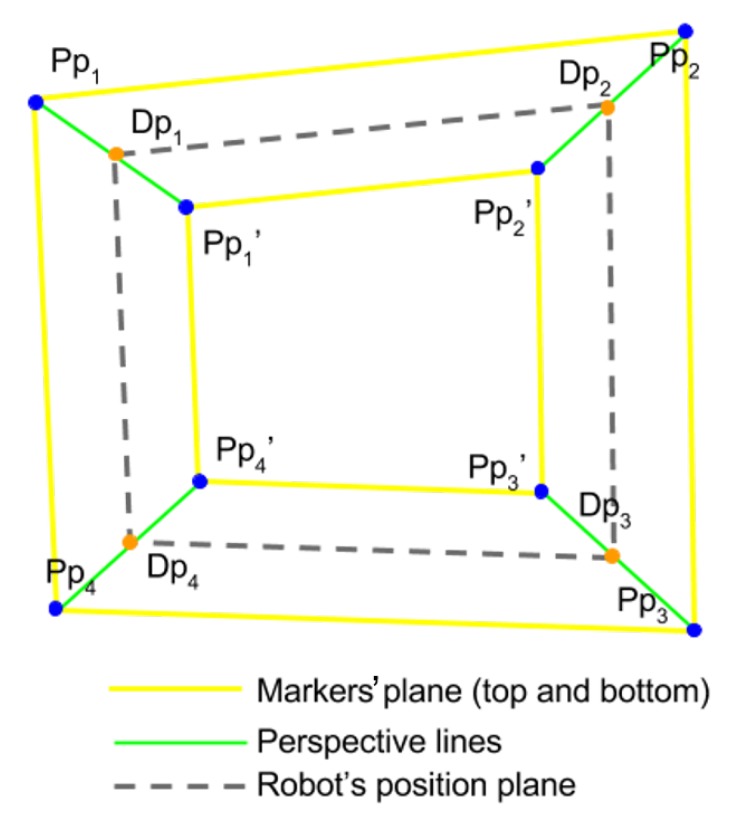
Diagram representing an image captured by the camera in which the top plane formed by the points $Pp_{n}$ and the bottom plane formed by $Pp_{n}^{\prime }$ points is highlighted in yellow. The $\vec{Pp_{n}Pp_{n}^{\prime }}$ are shown in green. Intersecting points of the robot’s submerged plane (grey dashed lines) are marked in orange.

To correct the scaling and perspective distortion, the localization system estimates the depth of the robot using the pressure sensor measurements and estimates the projection $Dp_{n}$ (orange dots in Figure 8) of it along the vertical edges of the perspective cube, represented by the green lines. This is currently achieved using a linear function, however further research is investigating non-linear approaches.

Once the depth of the plane in which the robot is submerged has been approximated, a matrix that describes the perspective transform is obtained using $Dp_{1-4}$ and the known distances between them, *a* and *b*. By multiplying each of the pixels of the image with this matrix, the perspective of the image will be corrected to make it look as if it were parallel to the camera.

The real-world position and attitude Rc=(X,Y,Z,θ) of the robot can be calculated by multiplying the same matrix to Pc. This gives the real-world position of the AVEXIS${}^{\mathrm{TM}}$ in the tank.

## 4. Integration of Sonar for 3D Reconstruction of the Environment

With an accurate position of the ROV, a 3D map of the underwater environment can be gathered through the sewing of multiple sonar “slices” together. This will enable the localization and characterization of fuel debris with accurate size, shape, and position of the debris, leading to efficient and safe decommissioning. The 3D reconstruction of the environment in a known test environment can also act as a verification of the localization system.

As discussed in Section 1, the IMAGENEX 831l pipe profiling sonar was used. Interface hardware was developed and placed within the AVEXIS${}^{\mathrm{TM}}$ with the sonar mounted beneath the ROV as seen in Figure 3.

**Figure 9:**
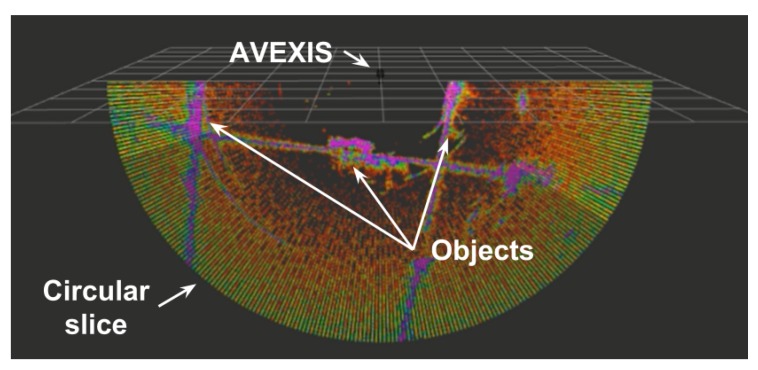
A 2D slice of the sonar during the technical demonstration at the Naraha Nuclear Test Facility.

The sonar was interfaced through a TCP/IP socket written in Python${}^{\mathrm{TM}}$ that collects, formats and outputs the data into a robot operating system (ROS) node. This node is combined with a 3D representation of the AVEXIS${}^{\mathrm{TM}}$ and the latest positioning data of the ROV to output a “sonar slice” to the RVIZ visualization package of ROS. The sonar outputs data in circular section slices, with each slice taking approximately three seconds, dependent on the survey distance. This can then be used to view the state of an unknown environment as well as mapping. Figure 9 shows a visualization of a single scanning slice, where the bright purple set of data points show the high intensity reflections incoming into the sonar and the green ones show lower intensities. A threshold can be applied to the incoming data to reduce the noise on the displayed data.

The 3D reconstruction of the environment was done by over imposing all the 2D sonar readings over a user-defined period of time whilst moving the AVEXIS${}^{\mathrm{TM}}$ within the environment. A complete 3D construction would take in the range of two minutes.

Each data point retrieved from the sonar was timestamped to ensure that it was synchronised with the localisation system and avoided placement of data in the wrong 3D position due to the movement of the ROV whilst a sonar slice was retrieved. For example, if the ROV rotated during the sonar data retrieval, the sonar slice was placed within the 3D reconstruction in a helix pattern.

## 5. External Vision Localization System Characterization

**Figure 10:**
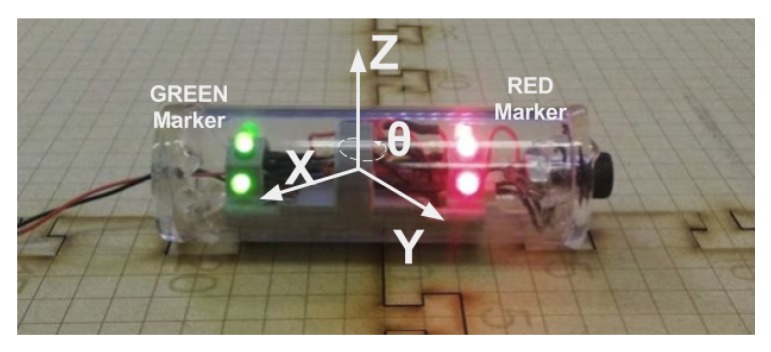
Miniature mock AVEXIS used for verification on grid.

To estimate the accuracy of the system across the FoV of the camera, an experiment was conducted where a scale version of the AVEXIS${}^{\mathrm{TM}}$ system was placed at different positions across a 1 m by 1 m grid layout and its position estimated. The AVEXIS${}^{\mathrm{TM}}$ was mounted on a fixed grid with defined spacings for validation as seen in Figure 10.

The system showed a mean absolute error of 5 mm across the test area, with the most accurate position to be the centre of the FoV (2.3 mm), and the worst accuracy to be in certain edges of the FoV (8.1 mm). The accuracies were not symmetrical about the camera’s central axis due to the light distribution during the test.

**Figure 11:**
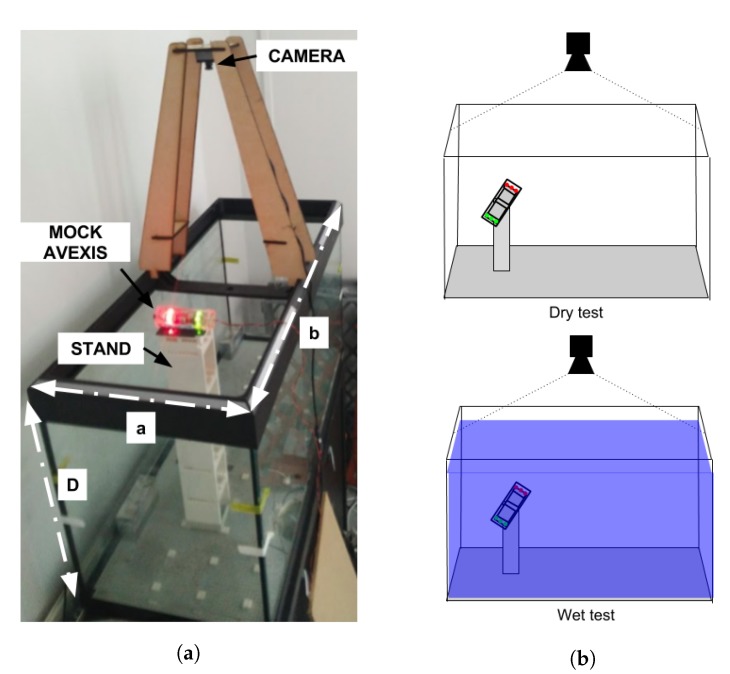
Experimental verification of mock AVEXIS^TM^ with Z-axis variation. The real-world test setup can be seen in (**a**), whilst (**b**) outlines that the test was completed both in a submerged and in-air environment.

The precision of the position and attitude estimation of the robot was then tested in a test tank using a stand to position the scale model of the AVEXIS${}^{\mathrm{TM}}$ in known positions along the *X*, *Y*, and *Z* axis. Thirty different positions were sampled in the *X* and *Y* plane at five different vertical distances to the camera. The experimental setup can be seen in Figure 11.

The standard deviation for the five samples at each of the 30 positions was then averaged for each vertical distance to the camera. The tests were performed without water (dry test) and with water (wet test).

The light conditions of the environment were set to avoid reflections of light sources to the camera, with the camera placed directly above the centre of the test tank. These are optimal conditions for image identification and the perspective algorithms. All the objects that could cause false positives were removed from the vicinity of the test tank.

The results of these tests are shown in Table 2, which shows that for the dry test the precision of the system is between 0.4 mm and 0.7 mm. Adding the water reduces the precision of the system to between 0.7 mm and 0.9 mm.

The *Z* precision was not calculated as it was determined by the physical mount as the mock AVEXIS${}^{\mathrm{TM}}$ does not contain a pressure sensor, therefore, it was a set input into the test setup. The accuracy of the depth sensor on the ROV is 10 mm.

## 6. Naraha Deployment

As mentioned in Section 2, the primary aim of the localization system was to help demonstrate the feasibility of identifying and localizing spent fuel debris using the AVEXIS${}^{\mathrm{TM}}$ vehicle and an imaging sonar at the Naraha Nuclear Test Facility.

**Figure 12:**
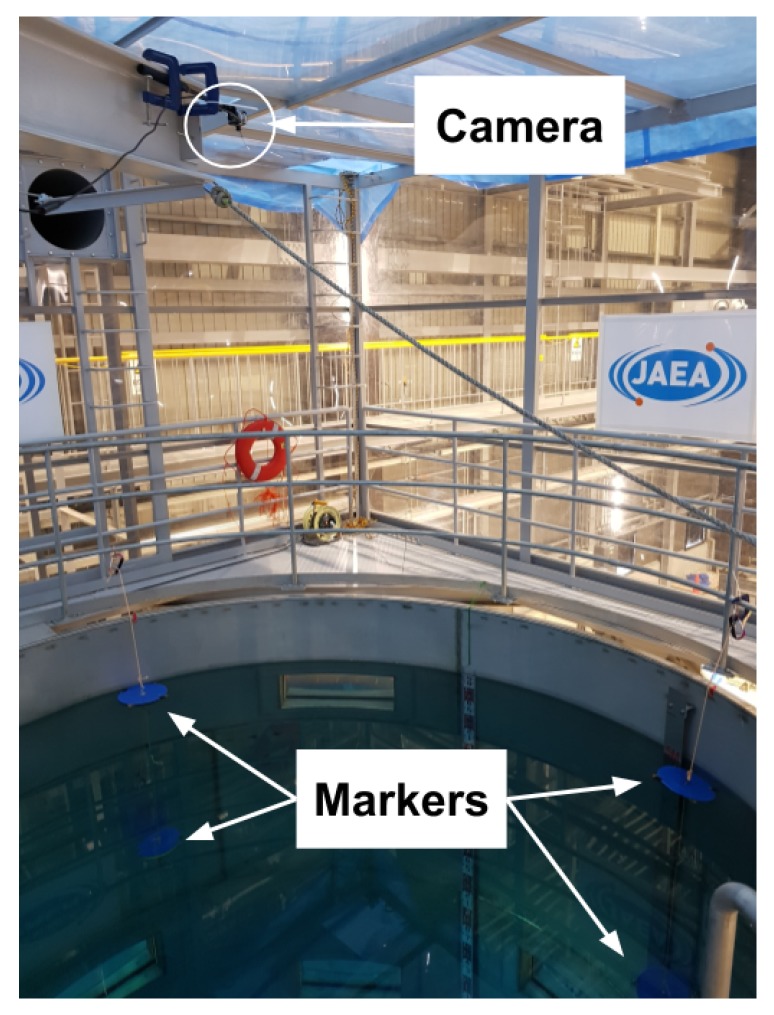
Setup of the camera in the Naraha Nuclear Test Facility.

The technical demonstration took place over 3 days with the camera mounted on an overhead crane giving a centred view of the working environment as seen in Figure 12.

**Figure 13:**
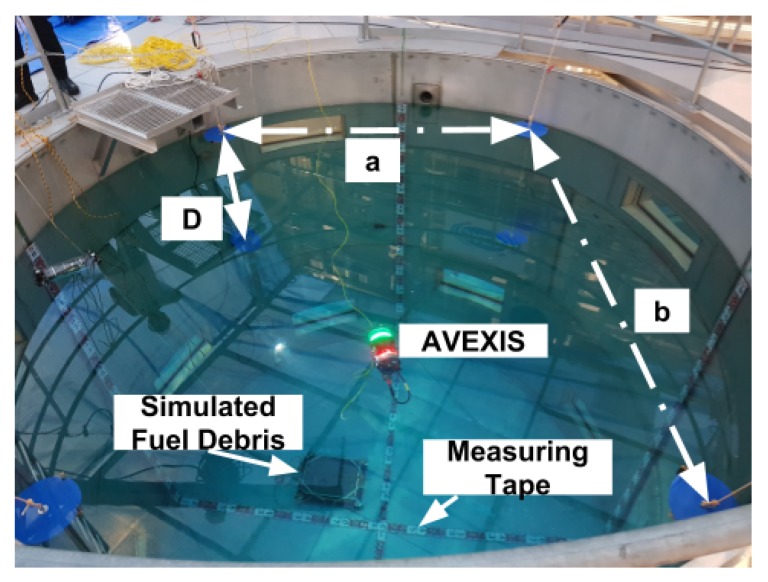
Setup of the markers in the Naraha Nuclear Test Facility. The four top markers (blue circles) can be seen at the top of the water and the bottom four markers can be seen at an offset of 1 m.

The markers for the $P_{n}$ and $P_{n}^{\prime }$ reference points, as seen in Figure 13, were blue circles made out of thin plastic with a diameter of 0.3 m and 2 m apart from each other, in the XY plane, and *D* was 1 m. Blue was arbitrarily chosen as the preferred colour for the passive markers. Any colour for the marker is suitable as the calibration procedure requires manual selection of the markers’ positions. In the chosen environment, the water had high clarity and therefore had little effect on distortion of the colour from the camera system.

The position of the large warehouse light sources in the environment created reflections on the surface of the water directed at the camera. This was mitigated by blocking and diffusing the ambient light to reduce the reflections into the camera. To assess the localization system’s positioning accuracy, measuring tape was mounted in the test tank, also seen in Figure 13 as the red and white lines crossing the tank.

## 7. Experimental Verification of the Complete System

**Figure 14:**
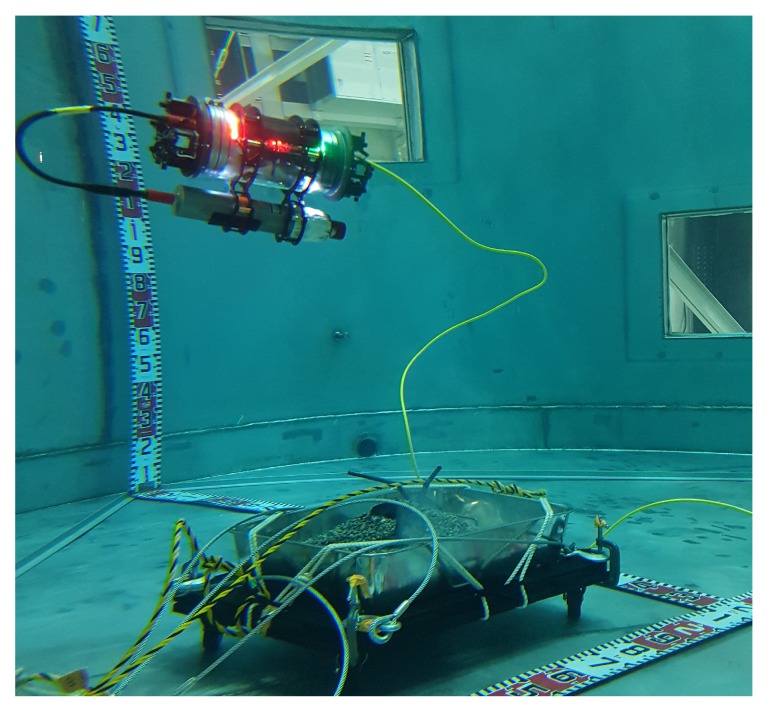
Deployment of the AVEXIS${}^{\mathrm{TM}}$ in the Naraha test tank, characterising the simulated fuel debris.

The technical demonstration began with visual inspection of the simulated fuel debris. The deployment of the complete ROV system within the Naraha tank for visual inspection can be seen in Figure 14.

**Figure 15:**
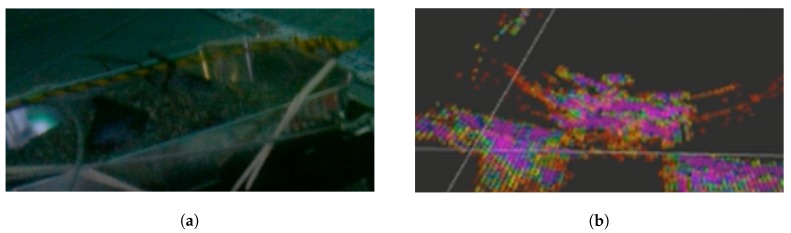
Comparison of an on-board image from the ROV of simulated debris (**a**) vs. one 2D slice of the 3D reconstruction from the sonar data (**b**).

After visual inspection, a 2D sonar slice was taken to determine the correct operation of the sonar and a broad overview of the simulated fuel debris. The general shape of the debris can be seen in Figure 15, compared with the camera view of the debris.

The sonar data can be interpreted as a dome of the debris at the correct size, however, although at specific angles of rotation, the debris is easily recognisable/comparable with a visual inspection, it is often difficult to highlight specific metallic blocks or pipes. This is due to only the cross-section being returned, with pipes particularly difficult to sense as sonar data could be returned as a “floating edge”. To take into consideration these difficulties in interpreting cross-sectional data and enable comparison with visual inspection, a 3D re-construction is required.

**Figure 16:**
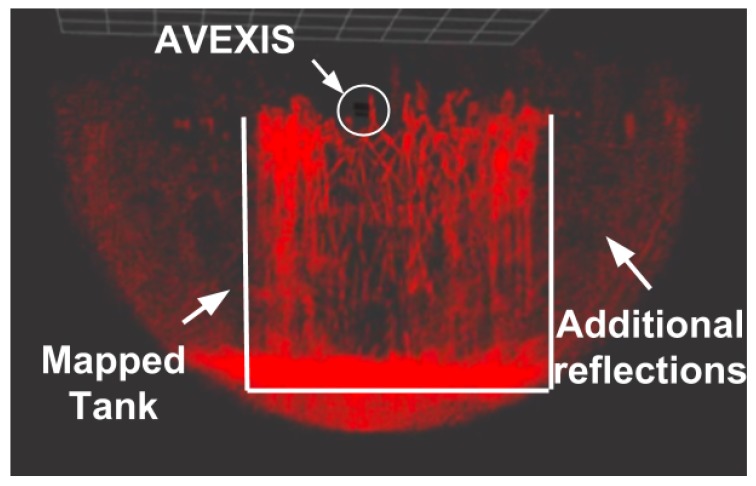
Output map showing the input from the sonar and positioning the robot on the output from the localization system.

After testing the functionality of the AVEXIS${}^{\mathrm{TM}}$, a series of tests to create a 3D reconstruction of the environment were conducted. Figure 16 shows an example visualization of a dataset of the deployment where the localization system returned an *x*, *y*, and *z* coordinate to combine with the sonar data.

Due to the orientation of the sonar, the “slices” of the environment are detected in a semi circle perpendicular to the AVEXIS${}^{\mathrm{TM}}$. This is highlighted by the noise returned at the extremes of the semi-circle slice in Figure 16.

In order to make a full map of the environment, the robot needs to rotate at least 180${}^{\circ }$, while moving slowly, in order for the sonar to return a recognisable 3D scan. To avoid noise on the pitch and roll axis, the AVEXIS${}^{\mathrm{TM}}$ was ballasted so that no movement could occur in the roll and pitch directions.

Complete verification of the system is only possible with the installation of a ground-truth system. Currently, these are prohibitively expensive, therefore, due to the size of the tank and inaccuracies in visual reading of the deployed measuring tape from a distance through the water, the exact accuracy of the localization system was difficult to verify. However, the output of a visibly recognisable 3D visualization and the position of the AVEXIS${}^{\mathrm{TM}}$ against the mounted measuring tape on the inner side of the tank suggested that the localization system had an accuracy of ∼100 mm. When combining the accuracy of the sonar (∼50 mm) with the positioning system accuracy, the 3D reconstruction had an overall accuracy of ∼150 mm.

## 8. Discussion

The use of a vision localization system has advantages and disadvantages. An accuracy of approximately 10 mm when the ROV is within 1–2 m, increasing to approximately 100 mm when the ROV is within 8 m, is more accurate than any deployable acoustic system and has a larger range than Radio-Frequency RF based systems. However, when the ROV moves further away from the camera, more of the light from the ROV is absorbed by the water and refracted at the air–water medium. This requires manipulation of the camera feed, for example, increased brightness, contrast, and exposure to enable tracking to continue. This is currently a manual process, which leads to the occasional loss of localization capability during settings adjustment.

Possible solutions to the difficulty of maintaining a strong tracked position at further distances could be to use larger and brighter active markers, to optimise the tracking algorithm to reduce false positives, and to move the camera to be within the water, removing difficulties with the change in medium.

A current limitation of the proposed solution is the requirement of passive land-markers to calibrate the system to a new environment where the distance between the camera and the water may vary. In harsh environments, this may not be feasible. However, moving the camera to be placed within the water and within a waterproof housing would remove the need for calibration markers as the vision system can be fully calibrated in a water tank prior to deployment. All that would be required during deployment would be a known position for the camera, which can then be defined within the program to output a global position.

Finally, the vision system may be affected by turbid water. It is believed that with bright enough markers, a reasonable position could still be obtained through averaging the returned markers. However, there may be increased error due to this process and also the refraction of light due to particulates in the water. This may also require recalibration of the localization system and is highlighted for future work.

The output 3D reconstruction showed considerable correlation with what was expected from visual inspection. However, as inaccuracies are present at each stage of the process from the sonar, to the localization system, a “domed” area of debris was witnessed with little clarity in individual metallic pipes or blocks. As acquiring sonar data is “slow” (approximately 3 s for one slice) the ROV would move during the slice with not all of the data points correctly positioned in the 3D reconstruction, which resulted in further errors during the visualisation of the data.

Adaptations to the data visualisation programming to remove returned acoustic noise, fusion of the data with other on-board systems such as an IMU for more accurate placement of sonar slices during rotation and exploration of alternative sonar systems could improve the returned 3D data that would be capable of highlighting specific metallic structures smaller than 100 mm.

With increases in localization accuracy, through increased camera resolution, automated exposure calibration and movement of the camera to within the water, we believe this could be a low cost deployable localization system that can be used in harsh environments such as Fukushima, but also nuclear storage tank facilities and tanks within the oil and gas industry.

## 9. Conclusions

The use of an overhead camera outside of the water, active markers on the robot, an on board pressure sensor, and known landmarks has been proven to be an accurate solution for underwater localization. This is the first external image-based localization system specifically designed to track underwater robots through an air/water interface that uses low cost off-the-shelf components (<$150 USD).

Robustness to the air/water interface, water ripple, light reflection, and alignment of the camera has been demonstrated, which makes the system easy to setup and able to operate even in environments with superficial currents. Although very bright spot-lights caused interference with the system, this is unlikely to be a problem in a test or deployment scenario as the light conditions and setup can be modified to suit the localization system.

The ability of the localization system to estimate the position with accuracy and precision has been demonstrated, with tests using several tanks and by tracking the AVEXIS${}^{\mathrm{TM}}$ vehicle while exploring the Naraha Nuclear Test Facility. The localization system made 3D image reconstruction of an unknown environment possible. An accurate representation of the tank has been reproduced with a broad overview of the debris returned. This enables the localization of debris and physical characterization of unknown environments.

The accuracy of the system might be increased by optimizing various stages of the system, from the marker design, background filtering, automatic aperture, and exposure compensation. If sufficient accuracy and precision are achieved this system could be used as ground truth for the development of micro-ROVs, micro-autonomous underwater vehicles and tROVs (tether-less ROVs) that require a positioning system in enclosed environments.

The system designed can be used to track any object that is within the FoV of the camera, provided that the required markers for the perspective cube are properly placed and there is line-of-sight between the tracked object and the camera. The tracking volume can be increased if there are more cameras, and their markers, are distributed around the environment with an overlap volume in the FoV of both cameras.

## Figures and Tables

**Table 1:** Summary of the most common underwater localization technologies against the low/moderate/high importance of possible characteristics of the environment. Also included for comparison are range, accuracy and costs.

	Acoustic	Electromagnetic (EM)	Vision	Inertial	Sonar	Lidar
**Visibility**	N/A	N/A	high	N/A	N/A	high
**Saliency**	N/A	N/A	moderate	N/A	high	high
**Line of sight**	high	moderate/high	high	N/A	N/A	N/A
**Conductivity**	N/A	high	N/A	N/A	N/A	N/A
**Latency**	high	low	moderate	low	high	moderate
**Range**	up to 10 km	0.05–1 m	vis. dependant	N/A	1–50 m	vis. dependant
**Accuracy**	0.5–5 m	1–5 cm	10–20 cm	1 cm–∞	5–100 cm	1 mm–5 cm
**Cost**	medium	low	low	low	high	very high

**Table 2:** Overall precision of the system at various distances to the camera during the wet and dry tests.

Distance from the Camera to the AVEXIS${}^{\mathrm{TM}}$	Dry Average Standard Deviation	Wet Average Standard Deviation
580 mm	±0.7 mm	±0.8 mm
680 mm	±0.5 mm	±0.8 mm
780 mm	±0.4 mm	±0.7 mm
880 mm	±0.4 mm	±0.8 mm
980 mm	±0.5 mm	±0.9 mm

## Abbreviations

The following abbreviations are used in this manuscript:

RPV	Reactor Pressure Vessel
PCV	Pressure Containment Vessel
ROV	Remotely Operated Vehicle
d.o.f	Degrees of Freedom
JAEA	Japan Atomic Energy Agency
NMRI	National Maritime Research Institute
UV	Underwater Vehicle
EM	Electromagnetic
RF	Radio Frequency
SLAM	Simultaneous Localization And Mapping
EVLS	External Vision Localization System
FoV	Field-of-view
ROS	Robot Operating System
